# The Book World of Medicine and Science

**Published:** 1893-06-17

**Authors:** 


### The Book World of Medicine and Science.

### FIRST IMPRESSIONS OF BOOKS RECEIVED.

[It is requested that all books intended to be noticed in this column may be sent to the Editor, at the Office, 428, Strand, W.C., not later than Tuesday evening in each week.]

The Physiology of the Senses. By John Gray M'Kendrick, M.D., LL.D., Professor of Physiology in the University of Glasgow; and William Snodgrass, M.A., M.B., Muirhead Demonstrator of Physiology in the University of Glasgow. With 127 illustrations. (London: John Murray, 1893.)

The University Extension Movement has taken firm root among us, and is accomplishing a great work in many large centres of population. The teaching has been thorough, and yet not over the heads of the scholars; and it has filled a wide gap in our education system. It has been necessary to provide a corresponding series of text books, more advanced than those used in schools, and not so erudite and full as those peculiarly suited to the most advanced students in the great seats of learning. The book before us is such a text book. It is written by accomplished physiologists thoroughly masters of their subject. It deals with rather recondite matters, but the treatment of them is comparatively simple and the subject is made plain to every intelligent reader. The book will be acceptable to a much wider circle than University Extension students. Medical students will find it very useful, and all who desire to understand something of the functions of the organs of sense will do well to take this book as their guide. The illustrations are admirable and numerous.

Chemistry for All; or, Alternative Elementary Chemistry in Accordance with the Syllabus of the Department of Science and Art. By W. Jerome Harrison, F.C.S., Chief Science Demonstrator for the Birmingham School Board; and Robert J. Bailey, Assistant Science Demonstrator. (London: Blackie and Son, 1893.)

This is one of Blackie's Science Text-Books, specially prepared for those working for the examination of the Science and Art Department. It seems to be admirably adapted for its purpose—terse, clear, and accurate. It is quite elementary in its scope, and devoid of chemical formulas and mathematical problems. It is nicely printed, and the illustrations are creditable.

Obstetric Aphorisms, for the Use of Students on Commencing Midwifery Practice. By Joseph Griffiths Swayne, M.D., Consulting Physician Accoucheur to the Bristol General Hospital. Tenth Edition. (London: J. and A. Churchill, 1893.)

When a students' manual has reached the tenth edition, it is needless to enlarge on its merits. Dr. Swayne's "Aphorisms" have met the needs of many generations of medical students and practitioners, and so far as it goes, it is an admirable work.

Outlines of the Diseases of Women. By John Phillips, M.A., M.D., F.R.C.P., Assistant Obstetric Physician to King's College Hospital, Examiner in Midwifery to the University of Glasgow, &c. With 120 illustrations. (London: Charles Griffin and Company, 1893.)

This is a text book for students and junior practitioners. The subject—than which none is more important for practitioners—is dealt with clinically and practically; there is no attempt to be exhaustive, but the author describes what he

THE BOOK WORLD OF MEDICINE AND SCIENCE.—(*continued*).

believes to be the best modes of treatment. Operations by abdominal section are not described. The illustrations are many of them taken from photographs, others are copied from different writers, and many are original diagrams. A short English Bibliography is found at the close of nearly every chapter. Dr. Phillips writes tersely and well, and his book will take a high place among its many rivals. Its teaching is sound.

A Medical Hand-book for the Use of Practitioners and Students. By R. S. Aitchison, M.B.C.M., F.R.C.P.E. With numerous illustrations. (London: Griffin and Co., Limited. 1893.)

This is a companion volume to Caird and Cathcart's "Surgical Hand-book." It is a very brief epitome of the larger text-books, and so will be of use to those who want to refer to a point quickly, or to those who want to furbish up for an examination a large area of work in a limited time. The book seems on the whole well done; it is nicely printed and got up.

Year-book of the Scientific and Learned Societies of Great Britain and Ireland. Comprising lists of the Papers read during 1892 before Societies engaged in fourteen departments of Research, with the names of their authors. Compiled from official sources. Tenth annual issue. (Charles Griffin and Co., Limited. London, 1893.)

The title of this work fully indicates its scope. It is a very useful book of reference.

### JUNE REVIEWS.

An eloquent plea for the Local Veto Bill is contributed to the Fortnightly Review by Archdeacon Farrar. He quotes a remarkable series of opinions from eminent men, both ancient and modern, testifying to the connection of drink with crime, disease, and misery. It is remarkable that in 1724 gin-drinking began to affect the masses, and in 1750 the London physicians drew up a memorial stating that there were then 14,000 cases of fatal illness due to gin alone. Such poisoning is not confined to one class of society only. Sir William Gull said before the Committee of the House of Lords that "a very large number of people in society are dying day by day, poisoned by alchohol, but not supposed to be poisoned by it." Mr. Mulhall, in his statistics, attributes to drink 48 per cent. of the idiocy in England. All the facts point to the urgent need in some shape or other for the regulation of the drink traffic, which in its present unrestrained form is able to render abortive every effort to raise the moral and physical well being of the people. Lady Jeune, in pleading the cause of the poor children's holiday, gives a necessary caution against spreading infection by omitting to procure a medical certificate in sending children to the country. "By applying at headquarters a certificate can always be procured to certify whether illness has been reported within a given time." The evil which a neglect of this simple precaution may entail is incalculable, and the cases of infectious outbreaks, reported from time to time as consequent on the exodus of so many thousand children yearly from London, tend to increase the already serious difficulty of placing them in satisfactory homes.

The Westminster Review has a fragmentary article in favour of cremation by the Rev. A. Newman, who leaves the question much where it was. It appears that in Germany it is greatly on the increase since, in addition to the crematorium established at Gotha in 1887, a new one has been set up and consecrated at Ohlsdorf, and a third at Carlsruhe was opened in October last.

